# Yellow fever resurgence: An avoidable crisis?

**DOI:** 10.1038/s41541-022-00552-3

**Published:** 2022-11-02

**Authors:** Nicole P. Lindsey, Jennifer Horton, Alan D. T. Barrett, Maurice Demanou, Thomas P. Monath, Oyewale Tomori, Michel Van Herp, Herve Zeller, Ibrahima Soce Fall, Laurence Cibrelus, J. Erin Staples

**Affiliations:** 1 Eliminate Yellow Fever Epidemic Risk Assessment Working Group, Geneva, Switzerland; 2 Arboviral Diseases Branch, U.S. Centers for Disease Control and Prevention, Fort Collins, CO USA; 3 Eliminate Yellow Fever Epidemics Strategy, High Impact Epidemics, WHO Health Emergencies Programme, World Health Organization, Geneva, Switzerland; 4 Department of Pathology and Sealy Institute for Vaccine Sciences, University of Texas Medical Branch, Galveston, TX USA; 5 Regional Office for Africa, World Health Organization, Ouagadougou, Burkina Faso; 6 Crozet BioPharma, Lexington, MA USA; 7 African Center for Genomics of Infectious Diseases (ACEGID), Redeemer’s University, Ede, Nigeria; 8 Médecins Sans Frontières, Brussels, Belgium; 9 Consultant, Paris, France; 10 WHO Health Emergencies Programme, World Health Organization Headquarters, Geneva, Switzerland

**Keywords:** Epidemiology, Viral infection

Yellow fever (YF), an acute viral hemorrhagic disease transmitted by infected mosquitoes, has the potential to spread rapidly and cause serious public health impact. The disease predominantly affects people in sub-Saharan Africa and tropical South America, where 40 countries are considered endemic and at high-risk for YF outbreaks^1^. Despite the availability of safe and effective vaccines since the 1930s, YF outbreaks continue to occur resulting in an estimated 109,000 severe cases and 51,000 deaths annually^2^. These figures are likely underestimates as most mild YF cases go undetected due to nonspecific symptoms and limited surveillance or laboratory diagnostic capacity in many at-risk regions.

Because of large explosive outbreaks in the last five years, YF has reemerged as a major international public health threat. In 2016, an explosive outbreak occurred in Angola, spreading to neighboring areas in the Democratic Republic of Congo and infecting expatriate workers, including at least 11 workers who returned to China while ill^3^. At the time of the outbreak in Angola, vaccination coverage and disease awareness were low as the last YF outbreak was in 1971. In addition, control measures, such as requiring a valid international certificate of vaccination for travelers, were not enforced^4^. Thirty million doses of YF vaccine were needed to stop the outbreak, which both outstripped the available global vaccine supply and led to the unprecedented use of fractional doses of the vaccine to prevent further disease spread^5^. In late 2016–2017, outbreaks of YF were also detected in coastal areas of Brazil where cases had not been reported since the 1940s and vaccination was not routinely recommended^6^. Again, fractional doses of the vaccine were needed to protect those residing in affected areas. Although fractional doses have been demonstrated to provide good short-term protection, questions remain if they will provide the same long-term protective immunity as a full dose^7–9^. Until these questions can be adequately answered, fractional doses should only be considered in emergency scenarios if there are insufficient doses of the vaccine to respond to active or imminent threats of large-scale amplification of YF^10,11^.

The YF outbreaks and resulting public health crises in Angola, the Democratic Republic of Congo, and Brazil underscored the need for a comprehensive, updated and intensified strategy to eliminate YF epidemics. To that end, the multi-partner global Eliminate Yellow Fever Epidemics (EYE) Strategy was established in 2017 to improve detection, outbreak preparedness, and response^1^. The EYE Strategy has 3 main objectives: (1) to protect at-risk populations through immunization; (2) to prevent international spread of the disease through vaccinating high-risk workers, enforcing International Health Regulations and supporting the development of resilient urban centers; and (3) to contain outbreaks rapidly by improving surveillance and timely access to validated diagnostic tests and emergency vaccines. The Strategy was endorsed by WHO’s Strategic Advisory Group of Experts on Immunization (SAGE) and launched in the African Region in 2018^1,12^. Through engagement with the EYE Strategy at country, regional and global levels in three regions (Africa, North Africa-Middle East, and the Americas), there have been remarkable achievements. Close to 250 million people have been vaccinated against YF in Africa since the inception of the EYE Strategy. In 2021 alone, more than 65 million people were vaccinated in Africa through routine immunization (RI) program (18 million) and preventive (43.7 million), reactive (3.8 million), and catch-up (1 million) campaigns^13^. Advocacy work by partner agencies has resulted in increased YF vaccine supply and improved confidence in production channels^14^. Twenty years ago, there were only 20 million doses of YF vaccine produced annually^15^. By 2016, the number of doses had increased to 93 million and over >150 million doses were available in both 2019 and 2020^15,16^. This improved supply has been instrumental to assure that surging demand for preventive mass vaccination campaigns (PMVCs) can be provided for in addition to the RI and outbreak response needs. The EYE Strategy has achieved stronger laboratory capacity for YF diagnostic testing, allowing for improved surveillance, faster diagnostic confirmations, and improving detection and response to outbreaks^17^. Innovative tools, such as simple-to-use national and subnational risk assessment tools, have been developed and implemented to allow for streamlined, transparent, and risk-informed vaccination allocations^13,18^.

**Fig. 1:**
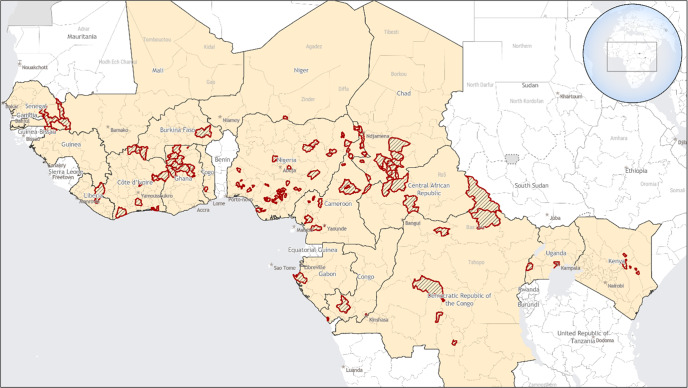
Yellow fever disease cases — Africa, September 2020-March 2022. Areas with confirmed and probable yellow fever disease cases are outlined in red.

Despite these successes, YF outbreaks continue to occur. In late 2020, confirmed YF outbreaks were reported in Nigeria, Senegal, and Guinea^19^. In 2021 and early 2022, 12 countries across Africa (Cameroon, Chad, Central African Republic, Côte d’Ivoire, the Democratic Republic of Congo, Gabon, Ghana, Kenya, Niger, Nigeria, Republic of Congo, and Uganda) reported confirmed YF outbreaks (Fig. 1)^20–22^. An additional five countries (Burkina Faso, Ethiopia, Liberia, Sierra Leone, and Togo) reported probable YF cases during the same time frame.

There are several factors that have likely contributed to the resurgence of YF in endemic areas. The global coronavirus disease-19 (COVID-19) pandemic and other events such as the Ebola outbreaks in West Africa, have had devasting impacts on healthcare delivery and access^23^. During the recent crises, RI programs have been unable to enhance the delivery of YF vaccines to children, resulting in declining RI coverages in west and central African countries with long-standing YF RI programs (72% in 2010 to 65% in 2020 and 2021)^24^. From 2020-2021, nine countries have an average YF RI coverage <60%, including Angola, Cameroon, Central African Republic, Chad, Democratic Republic of Congo, Equatorial Guinea, Gabon, Guinea, and Liberia^24^. Additionally, many affected countries are struggling with added vulnerabilities such as fragile governments and regional conflicts, that further limit capacities to prevent and respond to outbreaks and impede access to health services^20^. Finally, additional drivers of YF virus circulation, such as periodic increases in sylvatic transmission, climate and vector factors, and population movement likely have contributed to the recent increase^20,21,25^.

The specific factors that are driving the occurrence of YF cases vary by region and country. For instance, the decreasing RI coverage and the stress on existing health systems by competing public health priorities are likely important contributing factors to the increase in YF cases reported over the last two years in West and Central Africa^20^. Up until 2020, there had been minimal numbers of YF cases detected in West African countries following successful preventive mass vaccination campaigns previously conducted under the YF Initiative, the precursor to the EYE Strategy^26^. However, there have been several outbreaks detected since late 2020^19–22^. Field investigations conducted in Guinea determined that many of the cases were among children who had not been vaccinated in the RI program and were born after the last large preventive campaign^19^. In Nigeria, confirmed outbreaks were noted in six states (Enugu, Ebonyi, Delta, Benue, Bauchi, and Borno) that had not yet undergone preventive campaigns; some of the areas had been scheduled for campaigns but timelines were delayed due to COVID-19 and other public health crises. Levels of population immunity also have declined due to the movement of susceptible populations in affected areas^20^. The outbreak in Senegal was centered in communities located along the border where there was fluid population movement and recent interruptions to primary health services^19^. In Ghana, an investigation found that many of the affected persons belonged to nomadic communities who had not been vaccinated during previous campaigns, suggesting challenges in equitable or sustained access to vaccination services^20^. In East Africa, the resurgence of YF cases is likely because of increased sylvatic activity that occurs in the region usually after prolonged periods of inactivity and lack of adequate vaccination^25^. Several high-risk countries in Eastern Africa (Ethiopia, South Sudan, and Uganda) have yet to introduce YF vaccine into their national RI programs or scale up existing programs^19^.

The current resurgence of YF is of particular concern as increased population mobility in today’s world amplifies the likelihood of new introductions and resulting local transmission in large population centers near currently affected areas, like Abidjan or Lagos, or to other non-endemic countries with *Aedes aegypti* populations^27,28^. Many areas of the world without YF virus do have *Ae. aegypti*-borne diseases and could theoretically be at risk for YF outbreaks^3,4^. This global threat has been illustrated by the recent spread of similar *Ae. aegypti*-borne viruses including chikungunya, dengue, and Zika^29^.

Although YF vaccination protects against infection within 7–10 days, implementation of emergency vaccination campaigns to contain rapidly expanding outbreaks is often hampered by limited in-country vaccine supplies, challenges with timely requests to access the emergency stockpile, and problems with vaccine delivery^11,27^. In addition, there continue to be competing vaccination priorities (e.g., COVID-19, polio, measles vaccine campaigns) and limitations in YF vaccine supplies as one of the four WHO prequalified manufacturers has paused production during the refurbishing of their facility and a second manufacturer is facing potential supply chain issues. However, additional manufacturers expected to come online by 2030^30^. Effective YF control requires adequate and sustained population vaccination coverage. To achieve high vaccination coverage on a long-term basis, the optimal strategy is to incorporate YF vaccination into RI programs and implement supplementary immunization activities, including catch-up campaigns in older populations^1,28^.

If comprehensive healthcare services and immunization delivery continue to erode, we can expect more YF outbreaks to occur in additional countries where YF virus exists in sylvatic cycles, including in those with a history of previous campaigns where RI services are unable to sustain high vaccine coverages. Thus, it is of vital importance to close the existing immunity gaps to not lose what ground has been gained in the fight against YF. It is critical that the global public health community continue to provide essential support and encourage acceleration of these programs to protect against future outbreaks in established regions and prevent spread.
